# Disease Risk & Landscape Attributes of Tick-Borne *Borrelia* Pathogens in the San Francisco Bay Area, California

**DOI:** 10.1371/journal.pone.0134812

**Published:** 2015-08-19

**Authors:** Daniel J. Salkeld, Nathan C. Nieto, Patricia Carbajales-Dale, Michael Carbajales-Dale, Stephanie S. Cinkovich, Eric F. Lambin

**Affiliations:** 1 Department of Biology, Colorado State University, Fort Collins, Colorado, United States of America; 2 Woods Institute for the Environment, Stanford University, Stanford, California, United States of America; 3 Department of Biological Sciences, Northern Arizona University, Flagstaff, Arizona, United States of America; 4 Center of Excellence for Next Generation Computing, Clemson University, Clemson, South Carolina, United States of America; 5 College of Engineering & Science, Clemson University, Clemson, South Carolina, United States of America; 6 School of Earth, Energy and the Environment, Stanford University, Stanford, California, United States of America; University of Kentucky College of Medicine, UNITED STATES

## Abstract

Habitat heterogeneity influences pathogen ecology by affecting vector abundance and the reservoir host communities. We investigated spatial patterns of disease risk for two human pathogens in the *Borrelia* genus–*B*. *burgdorferi* and *B*. *miyamotoi*–that are transmitted by the western black-legged tick, *Ixodes pacificus*. We collected ticks (349 nymphs, 273 adults) at 20 sites in the San Francisco Bay Area, California, USA. Tick abundance, pathogen prevalence and density of infected nymphs varied widely across sites and habitat type, though nymphal western black-legged ticks were more frequently found, and were more abundant in coast live oak forest and desert/semi-desert scrub (dominated by California sagebrush) habitats. We observed *Borrelia* infections in ticks at all sites where we able to collect >10 ticks. The recently recognized human pathogen, *B*. *miyamotoi*, was observed at a higher prevalence (13/349 nymphs = 3.7%, 95% CI = 2.0–6.3; 5/273 adults = 1.8%, 95% CI = 0.6–4.2) than recent studies from nearby locations (Alameda County, east of the San Francisco Bay), demonstrating that tick-borne disease risk and ecology can vary substantially at small geographic scales, with consequences for public health and disease diagnosis.

## Introduction

Human infection by tick-borne pathogens is the culmination of interactions between the transmission biology of the pathogen, the ecology of the reservoir hosts and competent vectors, and the consequent exposure and disease in the human case. Local habitat and environmental conditions can influence any and all aspects of these interactions, and disease risk and incidence vary as a result [1,2].

California exhibits a high degree of climatic and habitat heterogeneity which influences tick abundance, reservoir host communities, and the entomologic risk of Lyme disease [1,2]. For example, hardwood-dominated woodlands exhibit higher densities of nymphal ticks infected by *Borrelia burgdorferi*, the etiologic agent of Lyme disease, than do conifer-dominated woodlands that include redwood or pine [2]. Human incidence also varies within the state, with most cases occurring in northwestern counties, and fewer cases being reported from southern California [3].

Given the habitat and climate diversity, it is unsurprising that recent research has highlighted a concurrent remarkable diversity of Californian tick-borne pathogens [4–6]. The western black-legged tick, *Ixodes pacificus*, is a known vector of *B*. *burgdorferi* sensu stricto (ss), but also harbors other *Borrelia* species, including *B*. *miyamotoi*, which has recently been recognized as a human pathogen [7–12]. Recent tick-borne pathogen surveillance observed eight borrelial genospecies in ticks and small mammals from just Alameda County, east of the San Francisco Bay [6]. Other emerging tick-borne pathogens in California include *Anaplasma phagocytophilum*, cause of human granulocytic anaplasmosis, and *Rickettsia* 364D which has caused clusters of eschar-associated illness [13–14].

Here we investigate the ecology of tick-borne pathogens in the San Francisco Bay Area of northern California. Specifically, we describe *Borrelia* ecology in recreational areas, and report variation in pathogen prevalence and western black-legged tick (*I*. *pacificus*) density. In addition, we investigate the importance of local landscape attributes–vegetation type, soil type etc.–upon tick-borne disease risk. Increased recognition of the ecology of these disease agents in an area of high human population density and significant outdoor use may facilitate adoption of preventive behaviors, public health and medical responses to tick-borne pathogens in California.

## Materials and Methods

### Study sites and tick collection

**Fig 1:**
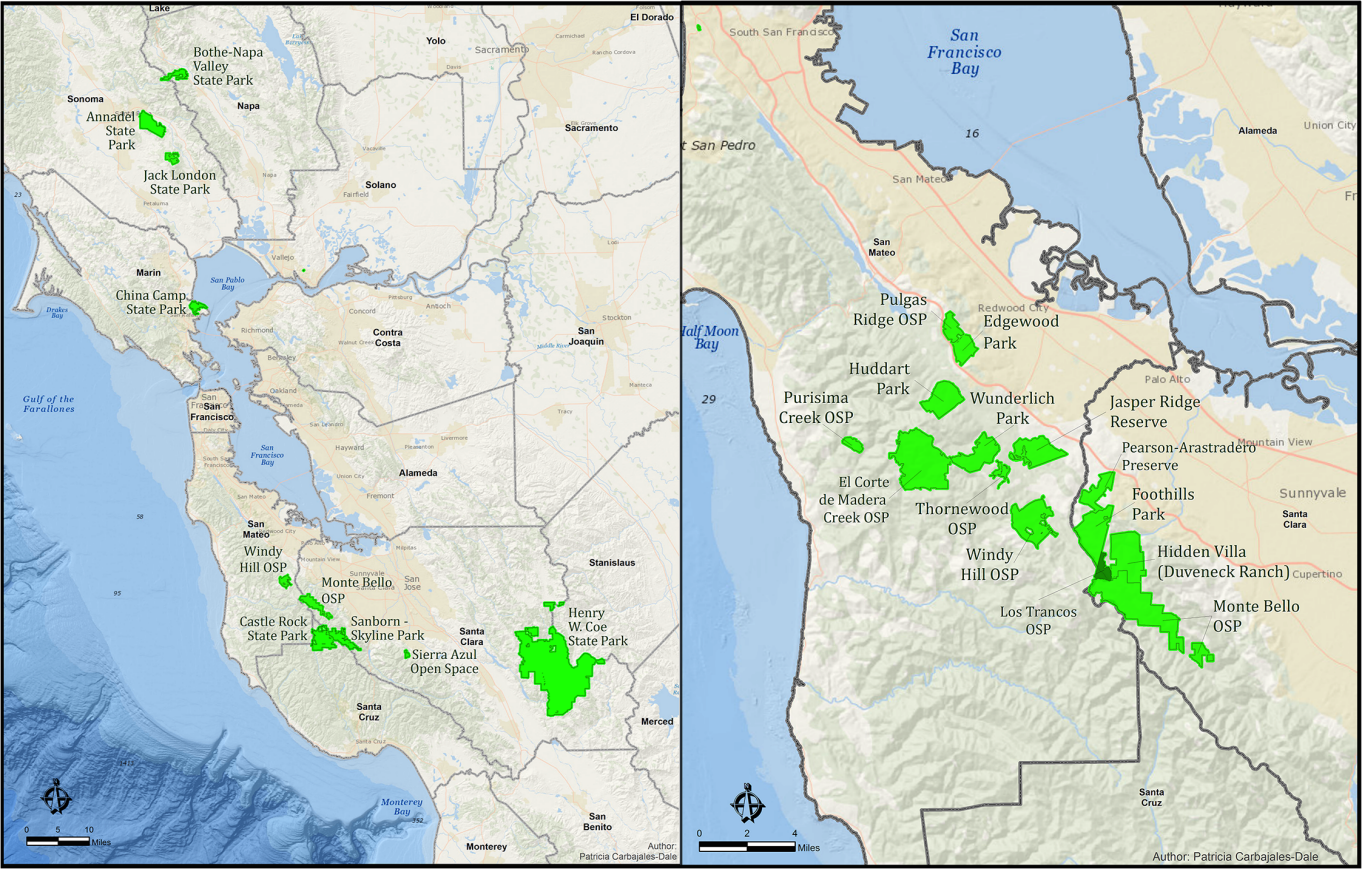
Recreational areas in the San Francisco Bay area (left) sampled for western black-legged ticks, with a close-up (right) of recreational areas sampled in San Mateo and Santa Clara Counties.

All sites (n = 20) were in the San Francisco Bay area (Marin, Napa, San Mateo, Santa Clara, Santa Cruz and Sonoma counties) and the majority were recreational areas (e.g., California State Parks (SP), Midpeninsular Open Space Preserves (OSP)) (Fig 1). We thank the Midpeninsular Regional Open Space District, San Mateo County Parks, Jasper Ridge Biological Preserve, City of Palo Alto, the Horse Park at Woodside, and California State Parks for permission to collect ticks. We also thank local community members of Portola Valley and Woodside and California Department of Public Health’s Vector-Borne Disease Section for assistance in collecting ticks.

In northern California, the period of highest nymphal abundance spans April to June, though local conditions dictate seasonal patterns of tick density and phenology [15]. For this study we collected ticks during May 2012 and May 2013 to ensure significant sample sizes of *I*. *pacificus* nymphs throughout the region. All ticks were identified to species and stage via morphology, and here we report studies of *I*. *pacificus* only. Nymphal and adult ticks were collected by dragging a 1m^2^ white flannel blanket along vegetation and/or leaf-litter. We recorded the number of nymphal ticks collected in 30m transects along trails. Transects were separated from each other by at least 60m to provide some degree of independence between transects, and each transect occurred in a single vegetation type. To augment sample sizes of ticks for data on pathogen prevalence, we collected ticks along trails between the 30m transects, but these samples are not included in the analyses of tick density-habitat relationships.

**Fig 2:**
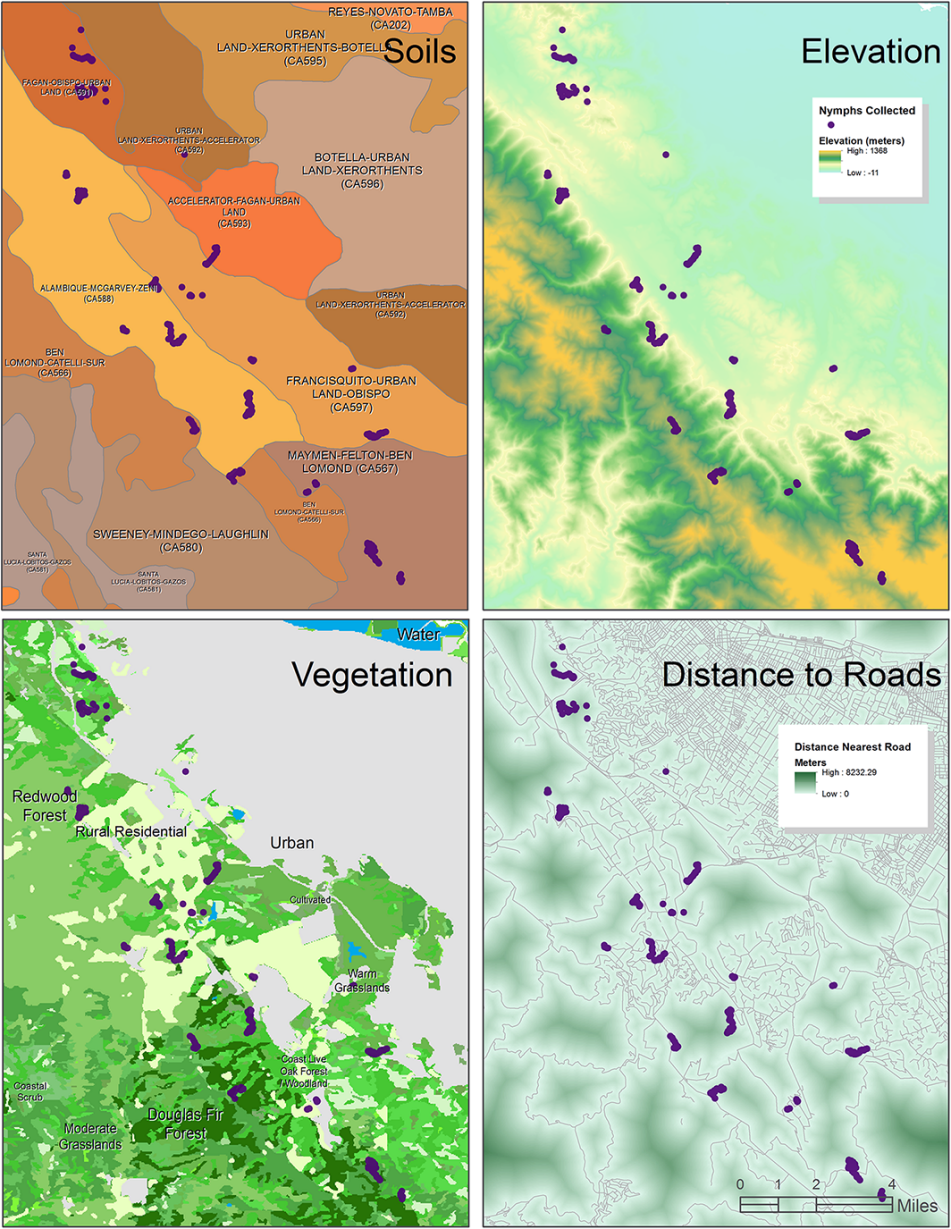
Study area showing independent variables: soil type (top left), elevation (top right), vegetation type (bottom left) and distance to roads (bottom right). Purple dots represent tick- drag transects in Santa Clara and San Mateo counties.

We recorded the GPS co-ordinates of the beginning of each transect to access GIS data on elevation (meters above sea level), distance to road (meters), and vegetation and soil type (Fig 2). GIS data were downloaded from the Conservation Lands Network (http://www.bayarealands.org/mapsdata.html). Elevation and distance to the nearest road are continuous variables; vegetation and soil types are categorical variables. For further details on vegetation and soil classification see [16–17]. We analyzed relationships between disease risk and landscape attributes only using data for which we had >15 transects for each vegetation classification. Habitat classifications included (1) coast live oak woodland–dominant species is coast live oak (*Quercus agrifolia*) with associated species including madrone (*Arbutus menziesii*), California blackberry (*Rubus ursinus*), and poison oak (*Toxicodendron diversilobum*); (2) redwood forest–dominant species is coastal redwood (*Sequoia sempervirens*) with associated species such as Douglas fir (*Pseudotsuga menziesii*) and tanoak (*Lithocarpus densiflorus*); (3) Douglas fir forest–dominant species is Pacific Douglas fir, with associated species such as coastal redwood, ponderosa pine (*Pinus ponderosa*), coast live oak and tanoak; (4) coastal scrub–dominant species are coyote brush (*Baccharis pilularis*) and poison oak, with associated species such as California sagebrush (*Artemisia californica*); (5) desert/semi-desert scrub–dominant species are California sagebrush and chamise (*Adenostoma* spp.) with other shrubs present; (6) warm grasslands–dominated by annual grasses and forbs, with varying amounts of native perennials, where July maximum temperatures are 26–30°C; and (7) moderate grasslands–dominated by annual grasses and forbs, with varying amounts of native perennials, where July maximum temperatures are 22–26°C. Sites often included multiple types of habitat, and habitat types were found across numerous recreational areas e.g., Windy Hill OSP contained coast live oak woodland, desert/semi-desert scrub, Douglas fir forest, coastal scrub, moderate grassland and warm grasslands.

### Detection of *Borrelia* spp. in *I*. *pacificus* nymphs

All ticks were removed from the site and stored in 70% ethanol. Nymphal ticks collected in 2013 were examined individually by a previously developed quantitative polymerase chain reaction (qPCR) diagnostic that identifies a segment of the 16S gene of *Borrelia* spp. DNA [18]. The assay utilizes primer pairs that can detect any member of the *Borrelia* genus and then combines species-specific hybridization probes to distinguish between Lyme group and TBRF group samples. DNA was extracted from individual ticks following manufacture’s protocols (DNeasy Blood and Tissue kit, Qiagen, Valencia CA) and stored at -20°C until molecular analysis. All diagnostic assays were performed using qPCR SsoFast Supermix 1X (Life Science Research, Bio-Rad, Hercules, CA) on a CFX96-Touch qPCR system (Life Science Research, Bio-Rad, Hercules, CA) and included three negative controls (3.6 μl water) on each run. Each 20 μl reaction contained primers at a concentration of 300 nM, probe at 200 nM (Applied Biosystems, Life Technologies, Carlsbad, CA), and followed a two-step protocol recommended by the manufacturer. Samples were considered positive if they had a cycle threshold (C_T_) value < 40 and logarithmic qPCR amplification plots. To identify *Borrelia* species and strain genotype we attempted to sequence the 16S-23S intergenic spacer (*rrs-rrlA*, IGS) of each qPCR-positive tick sample using a nested-PCR protocol [19]. The nested PCR product was further purified using the QIAquick kit (Qiagen, Valencia, CA) and then sequenced using capillary Sanger sequencing on an ABI 3730 sequencer with both forward and reverse primers (EnGGen, Northern Arizona University).

### Description of measures of entomologic risk

We report three measures of entomologic disease risk: (i) Density of nymphs (DON)—i.e., the number of nymphal ticks found per 100m of transect, for both 2012 and 2013; (ii) Infection prevalence of Borreliae, i.e., percentage of ticks testing positive for the disease agent for samples from 2013; (iii) Density of infected nymphs (DIN), calculated by multiplying nymphal infection prevalence by the density of nymphs, and reported as the density of infected nymphs/100m.

### Statistics and modeling approach

We constructed models to examine the influence of landscape variables (beginning with a full model incorporating elevation, distance from the nearest road, vegetation type, soil type and site) on the abundance of ticks in 30m-transects, and compared generalized linear models with Poisson errors and negative binomial errors to account for many zero-measures of ticks [20–21].

We compared the full model with simpler models and used Akaike’s Information Criterion (AIC) [22] to select the most parsimonious model with the highest predictive power of tick density. The model with the lowest AIC value was deemed the best, though other models within two AIC units were considered to be competing models with substantial support [23].

## Results

### Nymphal abundance

Nymphal *I*. *pacificus* abundance varied widely across all of our 20 study sites (0–10 nymphs/100m^2^; Table 1). Density of nymphs (DON) also ranged widely within recreational areas, depending on habitat or simply between different trails. For example, in Thornewood Open Space Preserve (OSP), DON was higher (3.1/100m^2^) in coast live oak forest compared to redwood habitats (1.2/100m^2^). In nearby Windy Hill OSP, DON varied between habitats dominated by coastal scrub and moderate grassland (0/100m^2^), and coast live oak forest at two locations: Betsy Crowder trail (10/100m^2^) and Meadow Trail, which also contained Douglas fir forest (1.25/100m^2^).

**Table 1:** Summary statistics of entomologic risk for *Ixodes pacificus* infected with *Borrelia* spp. in recreational areas of the San Francisco Bay Area. Data are presented on density of nymphal (DON) *I*. *pacificus* (i.e., number of nymphs/100m^2^ (calculated by (number of ticks collected/meters covered by transects) x100); density of infected nymphs (DIN) for *Borrelia* spp. (calculated by *Borrelia* prevalence x DON); and for *B*. *miyamotoi*, *B*. *burgdorferi* ss, and for *B*. *burgdorferi* sl.

Location	DON	DIN *Borrelia*	DIN *B*. *miyamotoi*	DIN *B*. *burgdorferi* ss	DIN *B*. *burgdorferi* sl
**Napa Co.**					
Bothe-Napa SP	0.8	0.04	0.02	0	0.02
**San Mateo Co.**					
Corte De Madera OSP	0.7	0	0	0	0
Edgewood Park	2.2	-	-	-	-
Horse Park at Woodside	0	0	0	0	0
Huddart Park	2.3	-	-	-	-
Jasper Ridge Biological Preserve	1.7	0	0	0	0
Los Trancos OSP	1.1	0.2	-	0.1	-
Pulgas Ridge OSP	1.0	0	0	0	0
Purisima Creek Redwood OSP	0	0	0	0	0
Thornewood OSP total	2.0	0.4	0.2	-	-
*Thornewood OSP*: *coast live oak forest*	3.1	0.3	-	-	-
*Thornewood OSP*: *redwood forest*	1.2	0.2	0.2	0	0
Windy Hill OSP total	5.2	0.6	0.2	0	0.1
*Windy Hill OSP–Betsy Crowder Trail* ^*1*^	10	1.0	0.4	-	0.2
*Windy Hill OSP–Anniversary Trail* ^*2*^	0	0	0	0	0
*Windy Hill OSP–Meadow Trail* ^*3*^	1.25	0.3	-	-	-
Wunderlich Co. Pk.	3.75	0.1	-	0.1	-
**Santa Clara Co.**					
Foothills Park	4.7	0.9	0.3	0.2	0.2
Hidden Villa	2.2	0.2	0.1	0.1	0.1
Monte Bello OSP	0.3	0.03	0.03	0	0
Pearson-Arastradero Preserve	0	0	0	0	0
Sierra Azul OSP	0.7	0	0	0	0
**Santa Cruz Co.**					
Castle Rock SP	0	0	0	0	0
**Sonoma Co.**					
Annadel SP	6	0.6	0.1	-	-
Jack London SP	0.7	0.1	-	0.04	-

^1^Betsy Crowder Trail is comprised of coast live oak woodland.

^2^Anniversary Trail is comprised of coastal scrub and moderate grassland.

^3^Meadow Trail is comprised of coast live oak woodland and Douglas fir forest.

We attempted to collect ticks from a total of 347 transects, and used data from 311 transects for analyses of landscape variables and entomologic risk (excluding 36 transects that occurred on private property, or in habitat types that were rare e.g., eucalyptus woodland). The most parsimonious model for explaining nymphal abundance included site, soil type, and distance to roads, with a negative binomial distribution. However, equivalent models within two AIC units contained vegetation and elevation, also with negative binomial distribution (Table 2). Nymphs were more frequently found in coast live oak woodland (40.4% of transects) and desert/semi-desert scrub (48.0% of transects) habitats (Table 3).

**Table 2:** Results of model selection to explain nymphal *I*. *pacificus* abundance.

Model	Model parameters	Df	Deviance	P	AIC
1	Site	19	64.0	<0.001	605.84
	Soil Type	6	23.9	<0.001	
	Distance to roads	1	4.6	0.033	
2	Site	19	67.0	<0.001	606.82
	Vegetation	6	21.4	0.002	
	Soil Type	6	14.4	0.026	
	Distance to roads	1	4.8	0.029	
	Elevation	1	2.8	0.093	
3	Site	19	66.7	<0.001	607.64
	Vegetation	6	21.3	0.002	
	Soil Type	6	10.7	0.026	
	Distance to roads	1	4.7	0.030	

**Table 3:** Habitat classifications and entomologic risk of *Borrelia*.

Habitat type	Number transects with *I*. *pacificus* nymphs/Number of transects	Abundance of nymphs/100m^2^ (number of nymphs/total meters)	Mean nymph abundance if present in transects (range)	*Borrelia* spp. prevalence^1^	*B*. *miyamotoi* prevalence	*B*. *burgdorferi* ss prevalence	*B*. *burgdorferi* sl prevalence^2^
Coast live oak woodland	42/102 (41.2%)	3.1 (95/3060)	2.3 (1–9)	N: 9/101 (8.9, 4.1–16.2) A: 3/42 (7.1, 1.5–19.5)	N: 4/101 (4.0, 1.1–9.8)		N: 1/101 (1.0, 0.02–5.4)
Coastal scrub	3/19 (15.8%)	0.7 (4/570)	1.3 (1–2)				
Desert/semi-desert scrub	12/25 (48.0%)	3.5 (26/750)	2.2 (1–5)				
Douglas fir forest	6/33 (18.2%)	0.9 (9/990)	1.5 (1–3)	N: 2/40 (5.0, 0.6–16.9) A: 3/26 (11.5, 2.4–30.2)	N: 1/40 (2.5, 0.06–13.2) A: 2/26 (7.7, 0.9–25.1)		N: 1/40 (2.5, 0.06–13.2) A: 1/26 (3.8, 0.1–19.6)
Moderate grassland	4/26 (15.4%)	1.2 (9/780)	2.3 (1–3)				
Redwood forest	18/73 (24.7%)	1.6 (35/2190)	1.9 (1–4)	N: 8/71 (11.3, 5.0–21.0) A: 5/43 (11.6, 3.9–25.1)	N: 3/71 (4.2, 0.9–11.9)	N: 3/71 (4.2, 0.9–11.9)	
Warm grassland	5/33 (15.2%)	0.5 (5/990)	1 (na)				

^1^Number ticks positive/Number ticks tested (% positive, 95% CI); N = nymphal *I*. *pacificus*; A = adult *I*. *pacificus*.

^2^Excluding *Borrelia burgdorferi* ss.

### *Borrelia*-infection prevalence

Overall, we found *Borrelia* spp. in 10.6% (37/349) of nymphs and 8.1% (22/273) of adults (Table 4). Of the 59 qPCR positive samples we were able to obtain IGS sequence data from 26 nymphal samples and 9 adult samples. We found *B*. *miyamotoi* in nymphal ticks at 8/16 sites, ranging in prevalence from 2.4–33.3% when present, and at a total prevalence of 3.7% (13/349) for all examined nymphs (Table 4). Prevalence of *B*. *miyamotoi* in adults was 3.9–9.1% when observed (3 sites), and 1.8% for all tested adults (5/273) (Table 4). The agent of Lyme disease, *B*. *burgdorferi* ss, occurred at NIPs of 3.6–7.4% when present (5/16 sites), and in 2.0% (7/349) of all nymphs (Table 4). For *B*. *burgdorferi* sl (excluding *B*. *burgdorferi* ss), NIP ranged from 2.0–3.7% at the four sites where it was detected, and was 1.7% (6/349) overall (Table 4). For adult ticks, *B*. *burgdorferi* sl prevalence was 3.8–10% at the two sites we found it, and was 0.7% (2/273) overall (Table 4).

**Table 4:** Numbers of *Ixodes pacificus* ticks positive for *Borrelia-*infection from recreational areas in the San Francisco Bay area in May 2013. Subtotals are also included for particular trails in Thornewood and Windy Hill OSPs to show within-site variation.

Location	Nymphs 2013				Adults 2013			
	*Borrelia spp*.^1^	*B miya*	*Bb ss*	*Bb sl*	*Borrelia spp*.	*B miya*	*Bb ss*	*Bbsl*
**Marin Co.**								
China Camp State Park (SP)	1/10 (10, 0.3–44.5)	1/10 (10, 0.3–44.5)			0/2			
**Napa Co.**								
Bothe-Napa SP	2/38 (5.3, 0.6–17.7)	1/38 (2.6, 0.1–13.8)		1/38 (2.6, 0.1–13.8)	3/26 (11.5, 2.4–30.1)	2/26 (7.7, 0.9–25.1)		1/26 (3.8, 0.1–19.6)
**San Mateo Co.**								
Corte De Madera Open Space Preserve (OSP)	0/6	0/6			0/3			
Jasper Ridge Biological Preserve	0/3				3/28 (10.7, 2.3–28.2)			
Los Trancos OSP	4/27 (14.8, 4.2–33.7)		2/27 (7.4, 0.9–24.3)		1/10 (10, 0.3–44.5)			1/10 (10, 0.3–44.5)
Pulgas Ridge OSP	0/6				1/11 (9.1, 0.2–41.3)			
Thornewood OSP total	5/28 (17.9, 6.0–36.9)	3/28 (10.7, 2.3–28.2)			1/29 (3.4, 0.1–17.8)			
*Thornewood OSP*: *Schilling Lake Trail (coast live oak woodland)*	2/19 (10.5, 1.3–33.1)				0/26			
*Thornewood OSP*: *Bridle Trail (redwood forest*)	3/9 (33.3, 7.5–70.1)	3/9 (33.3, 7.5–70.1)			1/3 (33.3, 0.8–90.6)			
Windy Hill OSP total	6/55 (10.9, 4.1–22.2)	2/55 (3.6, 0.4–12.5)		1/55 (1.8, 0.05–9.7)	4/25 (16.0, 4.5–36.1)	1/25 (4.0, 0.1–20.4)		
*Windy Hill OSP–Betsy Crowder Trail*	5/51 (9.8, 3.3–21.4)	2/51 (3.9, 0.5–13.5)		1/51 (2.0, 0.04–10.4)	2/4 (50.0, 6.8–93.2)			
*Windy Hill OSP–Anniversary Trail*					0/10			
*Windy Hill OSP–Meadow Trail*	1/4 (25.0, 0.6–80.6)				2/11 (18.2, 2.3–51.8)	1/11 (9.1, 0.2–41.3)		
Wunderlich Co. Pk.	2/27 (7.4, 0.9–24.3)		1/27 (3.7, 0.1–19.0)		0/22			
**Santa Clara Co.**								
Foothills Park	5/27 (18.5, 6.3–38.1)	2/27 (7.4, 0.9–24.3)	1/27 (3.7, 0.1–19.0)	1/27 (3.7, 0.1–19.0)	5/51 (9.8, 3.3–21.4)	2/51 (3.9, 0.5–13.5)	2/51 (3.9, 0.5–13.5)	
Hidden Villa	3/28 (10.7, 2.3–28.2)	1/28 (3.6, 0.1–18.3)	1/28 (3.6, 0.1–18.3)	1/28 (3.6, 0.1–18.3)	0/9			
Monte Bello OSP	2/20 (10, 1.2–31.7)	2/20 (10, 1.2–31.7)			0/37			
Sierra Azul OSP	0/2				0/1			
**Santa Cruz Co.**								
Castle Rock SP	0/2							
**Sonoma Co.**								
Annadel SP	4/41 (9.8, 2.7–23.1)	1/41 (2.4, 0.1–12.9)			0/4			
Jack London SP	3/29 (10.3, 2.2–27.4)		2/29 (6.9, 0.8–22.8)		4/15 (26.7, 7.8–55.1)			
**Total**	37/349 (10.6, 7.6–14.3)	13/349 (3.7, 2.0–6.3)	7/349 (2.0, 0.8–4.1)	6/349 (1.7, 0.6–3.7)	22/273 (8.1, 5.1–11.9)	5/273 (1.8, 0.6–4.2)	2/273 (0.7, 0.1–2.6)	2/273 (0.7, 0.1–2.6)

^1^Number positive/number tested (percentage positive, exact binomial 95% CI). Prevalence data for ticks categorized by habitat were difficult to determine, as many of the Borrelia-positive samples were from the ticks collected off-transect. By including ticks from recreational areas where the GIS-informed habitat type could be ascertained without ambiguity, we derived prevalence data for sufficient numbers of ticks from three vegetation types (Table 3). There were no significant differences between prevalence of infection with Borrelia spp. in the three vegetation types.

Prevalence data for ticks categorized by habitat were difficult to determine, as many of the *Borrelia*-positive samples were from the ticks collected off-transect. By including ticks from recreational areas where the GIS-informed habitat type could be ascertained without ambiguity, we derived prevalence data for sufficient numbers of ticks from three vegetation types (Table 3). There were no significant differences between prevalence of infection with *Borrelia* spp. in the three vegetation types.

### Density of infected nymphs (DIN)

Combining DON and NIP to generate DIN measures showed that exposure risk for tick-borne *Borrelia* spp. (number of *Borrelia-*infected nymphs/100m^2^) also varies widely, but was highest in transects that occurred in coast live oak-dominated woodlands, e.g., Windy Hill OSP–Betsy Crowder (1.0), Annadel State Park (0.6), Los Trancos OSP (0.5), and Foothills Park (0.4) (Table 1). Across all sites, DIN was largely equivalent for all three Borreliae detected on transects, though with idiosyncrasies. For example, in Thornewood OSP, tick density–and therefore sample size–was low in redwood habitat but *B*. *miyamotoi* NIP was high (33.3%), generating a DIN of 0.2/100m^2^. In contrast, in coast live oak woodland habitats within Thornewood OSP, nymphs were abundant (3.1/100m^2^, 4^th^ highest density) but we did not observe ticks infected with *B*. *miyamotoi*.

### Comparisons with other California *Borrelia* studies

The prevalence of *B*. *miyamotoi* is higher in our study area compared to nearby Alameda County [6], for both nymphal and adult ticks (Table 5). *B*. *burgdorferi* prevalence in nymphs was higher in Alameda County than in our study sites, though there was no appreciable difference in *B*. *burgdorferi* prevalence in adult ticks in both areas. It should be noted that sampling methods and PCR analyses may differ between our study and the study performed in Alameda County, and so these results should be interpreted with caution.

**Table 5:** Prevalence data for *Borrelia* in *I*. *pacificus* ticks from previous studies in California.

Location	Tick life stage	*B*. *miyamotoi* prevalence^1^	*B*. *burgdorferi* sl prevalence	*B*. *burgdorferi* ss prevalence	Reference
Bay Area	Nymphs	13/349 (3.7, 2.0–6.3)	11/349 (3.2, 1.6–5.6)	7/349 (2.0, 0.8–4.1)	This study
Bay Area	Adults	5/273 (1.8, 0.6–4.2)	4/273 (1.5, 0.4–3.7)	2/273 (0.7, 0.1–2.6)	This study
Bay Area	Adults	14/1108 (1.3, 0.7–2.1)	13/1108 (1.2, 0.6–2.0)	6/1108 (0.5, 0.2–1.2)	Salkeld et al. 2014
Alameda County	Nymphs	11/2890 (0.4, 0.2–0.7)	189/2890 (6.5, 5.6–7.5)	145/2890 (5.0, 4.3–5.9)	Fedorova et al. 2014
Alameda County	Adults	13/3070 (0.4, 0.2–0.7)	29/3070 (0.9, 0.6–1.4)	23/3070 (0.7, 0.4–1.1)	Fedorova et al. 2014
Mendocino Co.	Nymphs			264/5431 (4.9, 4.3–5.5)	Eisen et al. 2010

^1^Number positive/number tested (percentage positive, 95% CI).

Prevalence of Borreliae did not differ in adult ticks that were collected in 2013 (this study) and 2012 [12] in similar areas (*B*. *miyamotoi*: χ^2^ = 0.19, p = 0.67; *B*. *burgdorferi* ss: χ^2^ = 0.005, p = 0.94; *B*. *burgdorferi* sl: χ^2^ = 0.007, p = 0.93). *B*. *burgdorferi* prevalence was lower for nymphs in this study when compared to nymphs with *B*. *burgdorferi* ss in Mendocino County [2].

## Discussion

Our tick surveillance data echo recent studies that show that *Borrelia* spp. are ubiquitous in the Bay Area where western black-legged ticks are abundant [6, 11–12, 24]. Our model linking tick abundance with landscape attributes did reveal a few significant statistical associations. Vegetation type, soil type, site and distance to roads were found to be significant factors influencing nymphal abundance. However, there were no significant differences between prevalence of infection with *Borrelia* spp. in different vegetation types. Exposure risk for tick-borne *Borrelia* spp. varied widely across the landscape, but was highest in coast live oak-dominated woodlands. These spatial associations are likely too weak to serve as a basis for a spatial targeting of preventive public health policies and information campaigns for recreational areas in the Bay Area. A larger sample size and repeated sampling over the season and over multiple years would be required to support such policies. However, these results do suggest that tick abundance may be idiosyncratic with respect to particular recreational areas. A locally-specific knowledge of local risk of tick exposure could promote tick-borne disease awareness and appropriate preventative measures.

The recent revelation that *B*. *miyamotoi* can cause disease in humans [7–8, 10] has prompted increased surveillance for this pathogen in tick populations. Adult ticks exhibited a *B*. *miyamotoi* prevalence of 1.8% (5/273 adults), which is similar with adult ticks sampled from many of the same sites in 2012 (1.3%; 14/1108 ticks) [12]. The 2012 prevalence was garnered by testing pooled samples of adult ticks, and therefore is a minimum estimate of prevalence, and may account for the slightly lower prevalence estimates from 2012.

Our data for recreational areas in the San Francisco Bay Area shows that *B*. *miyamotoi* prevalence in nymphs and adults is roughly equivalent to that of *B*. *burgdorferi* sensu lato (sl). This is in contrast to other regions in the United States—e.g., *B*. *burgdorferi* is the dominant spirochete within nymphal *I*. *scapularis* in the north-eastern US [25]. Similarly, in Sweden and Japan, *B*. *miyamotoi* is less commonly found than other Borreliae that cause Lyme-borreliosis—i.e., *B*. *burgdorferi*, *B*. *garinii* and *B*. *afzelii* [26–27]. Even at a more local scale, *B*. *miyamotoi* prevalence is higher in our study sites compared to ticks in nearby Alameda County [6].

We cannot yet explain why there is such an equivalent rate of *B*. *miyamotoi* and *B*. *burgdorferi* infection in *I*. *pacificus* ticks in our study area of California, though we surmise that factors such as reservoir host ecology, tick abundance and phenology, transmission dynamics and habitat heterogeneity may all have some influence. For example, horizontal transmission of *B*. *miyamotoi* can occur during co-feeding of naïve larvae and infected nymphs (*I*. *scapularis*), and subsequently for at least three weeks [25]. The simultaneous questing activity of *I*. *pacificus* larvae and nymphs in California, in comparison with a more asynchronous phenology of *I*. *scapularis* larvae and nymphs in the north-east, hypothetically allows the potential for increased transmission of *B*. *miyamotoi* during co-feeding and may account for higher relative abundance of in the Bay area. It is important to note that, unlike *B*. *burgdorferi*, *B*. *miyamotoi* can be transmitted trans-ovarially [25]. Furthermore, vertebrate hosts may differ in abundance and reservoir potential (the ability to infect feeding ticks) compared to areas that have received more attention e.g., Mendocino County where western gray-squirrels are one of the predominant reservoir hosts of *B*. *burgdorferi* ss [1, 28–29].

Density of nymphal ticks (DIN) varied widely, and when combined with data on the prevalence of pathogens suggests that disease risk varies widely. Reports on density of infected nymphs are not as common as reports of pathogen prevalence, so generalizations are difficult to make. In Mendocino County, densities of *B*. *burgdorferi* ss infected *I*. *pacificus* nymphs from 0–2.04/100m^2,^ with a median density of 0.06/100m^2^ [2]. Estimates of DIN from our study are lower than the Mendocino County ranges: 0–1.0 when considering *Borrelia*, but smaller values for *B*. *burgdorferi*. This may be attributable to higher densities of ticks in Mendocino County. This emphasizes the point that multiple measures of disease risk need to be gathered [30].

With regards to bites by western black-legged ticks in the San Francisco Bay area, *B*. *miyamotoi* potentially poses as much risk of transmission to humans as *B*. *burgdorferi*. We are unaware of any reports documenting human incidence rates of *B*. *miyamotoi* in California, and this remains an unknown public health concern. In the north-east, seroprevalence rates (using IgG antibodies) in human populations ranges from 3.6% to 9.8% depending on specifics of the subject group e.g., within a study of 639 heathy participants from Rhode Island and Massachusetts, seroprevalence of *B*. *miyamotoi* infection was 3.9% (compared to 9.4% for *B*. *burgdorferi*) [31]. Thus, *B*. *miyamotoi* infection is not a rare infection in the northeastern United States [32].

In these areas of the northeastern United States where human seroprevalence has been examined, rates of *B*. *miyamotoi* infection in nymphal *I*. *scapularis* ticks are low: 1.1–1.6% in Rhode Island, and 2.5% in Massachusetts [18, 25]. Tick densities of 4.9 (SD = 23.5) nymphs per 100 m transect have been reported from Block Island in Rhode Island state [33]. By comparison, in our California study sites, nymphal infection prevalence of *B*. *miyamotoi* in our California study sites is higher (3.8% overall), though nymphal tick densities tend to be lower (Table 2).

Diagnosis of *Borrelia miyamotoi* disease can be complicated. *B*. *miyamotoi* infections may be misdiagnosed (e.g., for human granulocytic anaplasmosis) if the diagnosis is based only on clinical examinations, and not confirmed by specific laboratory assays [34]. Additionally, there may be previous exposure to *B*. *burgdorferi*, which can complicate interpretations of diagnostic tests [32]. Nonetheless, further research is needed to address the potential incidence of *B*. *miyamotoi* disease in human populations exposed to this pathogen in California, especially in areas where *B*. *miyamotoi* prevalence in tick populations is relatively high.

Overall, our data demonstrate that the ecology of *Borrelia* pathogens in California is highly variable at small geographical scales. Consequently, public health agencies and physicians should recognize that diverse disease risk and pathogen ecology may culminate in tick-borne diseases that demonstrate symptoms that are not entirely consistent with infections with *B*. *burgdorferi* s.s. Similarly, our results illustrate western black-legged ticks and their pathogens are ubiquitous in the San Francisco Bay Area, and therefore the general public should incorporate tick-bite prevention procedures when pursuing recreational activities in the locality.
